# Importance of Particle Size-Fraction Analysis in Suspensions

**DOI:** 10.1289/ehp.1002354

**Published:** 2010-09

**Authors:** Loretta Müller, Peter Gehr, Andreas C. R. Mayer

**Affiliations:** Institute of Anatomy, Division of Histology, University of Bern, Bern, Switzerland, E-mail: loretta.mueller@ana.unibe.ch; Technik Thermische Maschinen Niederrohrdorf, Switzerland

We would like to comment on the article by Cho et al. (2009), which was published in the November 2009 issue of *Environmental Health Perspectives* (*EHP*). We read the paper with great interest because the size-dependent effects of particulate matter are very important but have not yet been definitively clarified. In reporting the size-dependent effects of particles, it is essential to know the size distributions of the applied particle solutions, as well as the specific particle size fractions administered.

Cho et al. (2009) did not use direct inhalation exposure, which is the most relevant exposure route for airborne particles (Oberdörster et al. 2005). The particulate matter was sampled, resuspended in methanol and saline, and administered via pharyngeal aspiration to mice (50 μL saline containing 25 or 100 μg particulate matter). Because of the effort required for inhalation studies with size-fractionated airborne particulate matter, the particles were collected and resuspended. However, it is regrettable that the authors did not analyze the size fraction in the suspensions. Particles react in suspensions, especially by forming aggregates, and the reactions are dependent upon the specific composition of the suspension liquid (Teeguarden et al. 2007). Therefore, we consider it a significant drawback of the study of Cho et al. (2009) that they made statements concerning particulate size effects without taking into consideration the characteristics of the particle suspension used for aspiration. This should have been clearly addressed in the “Discussion” of their article. We are surprised that the reviewers did not highlight this point.

We are interested in studies that take into account the size effects of particles, both environmental and engineered, and that consider both direct exposure and other modes of exposure. We hope that *EHP* will take this into consideration during the peer-review process in the future.
